# No Effect of NGAL/lipocalin-2 on Aggressiveness of Cancer in the *MMTV-PyMT*/FVB/N Mouse Model for Breast Cancer

**DOI:** 10.1371/journal.pone.0039646

**Published:** 2012-06-21

**Authors:** Elisabeth P. Cramer, Andreas Glenthøj, Mattias Häger, Anna Juncker-Jensen, Lars H. Engelholm, Eric Santoni-Rugiu, Leif R. Lund, Ole D. Laerum, Jack B. Cowland, Niels Borregaard

**Affiliations:** 1 The Granulocyte Research Laboratory, Department of Hematology, Copenhagen University Hospital, Copenhagen, Denmark; 2 Finsen Laboratory, National University Hospital, Rigshospitalet, University of Copenhagen, Denmark; 3 Department of Pathology, National University Hospital, Rigshospitalet, University of Copenhagen, Denmark; 4 Department of Cellular and Molecular Medicine, University of Copenhagen, Denmark; 5 The Gade Institute, Section of Pathology, University of Bergen, Norway; University Medical Center, The Netherlands

## Abstract

NGAL/lipocalin-2 is a siderophore-binding protein that is highly expressed in several cancers. It is suggested to confer a proliferative advantage to cancer cells. Its expression has been correlated with aggressiveness of breast cancer as determined both in patients and in mouse breast cancer models. This was recently confirmed in two mouse models of spontaneous breast cancer in wild-type and lipocalin-2-deficient mice. We used a similar strategy using a different mouse strain. Lipocalin-2-deficient mice and mouse mammary tumor virus-polyoma middle T antigen (*MMTV-PyMT*) mice were crossed into the same FVB/N background. All mice developed tumors by week 8. The mice were sacrificed on week 13 and tissue was processed for biochemical and histological analysis. The total tumor volume and number of metastases were quantitated in 26 lipocalin-2-deficient mice and 34 wild-type controls. Lipocalin-2 expression in tumors of *MMTV-PyMT*-positive and wild-type mice was assessed by quantitative real-time PCR and by immunohistochemistry. The expression of the lipocalin-2 receptors 24p3R and megalin and of Mmp-9, transferrin receptor, and Bdh2 (a producer of a mammalian siderophore) were quantitated by real-time PCR. No significant difference was observed between wild-type and lipocalin-2-deficient mice. Lipocalin-2 was highly expressed in tumors from wild-type mice, but the expression did not correlate with tumor size. No effect of lipocalin-2 was observed with respect to time to tumor appearance, total tumor volume, or to the number of metastases. Histology and gelatinolytic activity of the mammary tumors did not differ between wild-type and lipocalin-2-deficient mice. We conclude that NGAL/lipocalin-2 does not invariably affect the aggressiveness of breast cancers as assessed in mouse models, thus questioning the role of lipocalin-2 in cancer development.

## Introduction

NGAL, neutrophil gelatinase associated lipocalin, was so named by its discoverers as a lipocalin present in human neutrophil specific granules, in part covalently associated with gelatinase B/matrix metalloproteinase-9 (MMP-9) [1], [2]. Highly homologous proteins are found in other species such as exFAB in fowls [3] and neu-related lipocalin induced in rat mammary tumors by the *neu* oncogene [4]. The mouse orthologue was identified as an oncogene and termed 24p3 [5] but has also been termed major urinary protein [6] and siderocalin [7]. These different names are united in the term lipocalin-2 encoded by the *LCN2/Lcn2* gene in man and mouse, respectively.

Soon after the discovery of lipocalin-2 as a constituent of neutrophil specific granules, lipocalin-2 was found to be highly up-regulated in epithelial cells at sites of inflammation [8]–[10] and to be highly expressed in some cancers [4], [11]–[13]. Lipocalin-2 has also received significant attention as an early and sensitive marker of damage to kidney tubule epithelial cells [14]–[17]. A function of lipocalin-2 in the innate immune defense against bacteria was demonstrated shortly after the discovery of lipocalin-2 as a siderophore-binding protein [7], [18], [19], as mice deficient in lipocalin-2 are more susceptible to infections by *E. coli* [20]–[22], *K. pneumonia* [23], and *M. tuberculosis* [24], [25] than their wild-type litter controls.

Lipocalin-2 has been inferred as an important regulator of apoptosis in myeloid cells [26]. This is claimed to depend on the ability of cells to take up lipocalin-2 via the 24p3-receptor [27]. Such uptake would result in apoptosis if lipocalin-2 is iron-deplete and promote growth if lipocalin-2 is iron-replete. The bacterial enzyme Ent A synthesizes the siderophore constituent 2,3-dihydroxy benzoic acid (DHBA). A mammalian homologue named BDH2 was recently described which generates 2,5-DHBA, the long sought for endogenous siderophore responsible for lipocalin-2 mediated iron trafficking in mammalian cells [28]. Catechol, a metabolite of tyrosine and other organic compounds, was identified as an alternative endogenous siderophore candidate [29]. The role of lipocalin-2 in controlling myeloid cell apoptosis is, however, questioned by the lack of obvious alterations in myelopoiesis of lipocalin-2-deficient mice [21] and by *in vitro* studies of the effect of lipocalin-2 on isolated human myeloid cells [30].

The involvement of lipocalin-2 in tumor genesis has particularly been studied in breast cancer, where lipocalin-2 (NGAL) expression is associated with poor prognosis in human primary breast cancer [31] and an increased urinary level of lipocalin-2 correlates with aggressiveness of the cancer [32]. The proposed mechanisms by which lipocalin-2 promotes growth and metastasis of breast cancer cells are multiple. Association of lipocalin-2 with MMP-9 was shown to induce allosteric activation of the enzymatic activity of MMP-9 and to protect MMP-9 from autodegradation [33], [34]. Lipocalin-2 has also been inferred as a direct regulator of expression of pro-oncogenic factors necessary for mesenchymal transition [32].

In order to directly address the role of lipocalin-2 in development of breast cancer, we initiated a study examining the role of lipocalin-2 in the spontaneous mouse mammary tumor virus-polyoma middle T antigen (*MMTV-PyMT*) mouse model. Since the genetic background influences the development of tumor growth and metastasis in *MMTV-PyMT* transgenic mice, we chose to use highly inbred mice from the FVB/N strain and heterozygous breeding to generate minimal genetic variation in order to detect even minor effects of lipocalin-2 in cancer development, a model that has been very useful in previous studies [35]–[37]. While our study was ongoing, two reports have appeared using the same or similar strategies [38], [39]. While major differences in the role of lipocalin-2 on tumor progression and metastasis was observed in these two studies, both confirmed that lipocalin-2 is associated with tumor progression and that complex formation with MMP-9 seems to be part of the mechanism. Our study used the same *MMTV-PyMT* mouse model as Berger et al. [38], but the results differ substantially, as we do not observe any significant effect of lipocalin-2 on any parameter associated with tumor growth and metastasis despite a brisk up-regulation of lipocalin-2 in the breast cancer cells. In addition, our study addresses whether the lipocalin-2 receptors and Bdh2 are expressed in the tumors.

## Materials and Methods

### Ethics statement

All animal experiments were conducted at The Department of Experimental Medicine, University of Copenhagen and National University Hospital, Rigshospitalet, Copenhagen, Denmark, in accordance with both institutional and national guidelines (Danish Animal Experiments Inspectorate, permission number 2007/561–1353). The review board at the Faculty of Health Science, University of Copenhagen, approved this study (P0599). An observer unaware of the genotypes of the mice performed all experimental evaluations. The mice were inspected daily and palpated once a week. No experiments were performed on live mice. Humane endpoint was set at tumor size influencing the general well being or behavior of the mice.

### Mice breeding

Congenic heterozygous male FVB/N-*MMTV-PyMT* mice were mated with heterozygous female *Lcn2* knock-out mice (*Lcn2*+/−) [20] that had been back-crossed to the FVB/N strain for 8 generations (N8). Their male FVB/N-*PyMT, Lcn2*+/− offspring (F1) were mated with FVB/N-*Lcn2*+/− (N9) females to generate the *PyMT, Lcn2*+/+ (n = 34); *PyMT, Lcn2*−/− (n = 26); *Lcn2*+/+ (n = 5), and *Lcn2*−/− (n = 7) mice used throughout the study (Figure S1).

### Quantification of primary tumors

Mice were examined weekly for mammary tumor onset by palpation for nodules in all 10 mammary glands. Tumor volume was assessed by measuring the length (*L*) and width (*W*) of individual tumors with a caliper and calculated by the formula V(tumor) =  π *L W* ^2^/6 [40]. The individual tumor volumes were summed to give the total tumor volume in each mouse.

### Tissue processing

Mice were anaesthetized by intraperitoneal administration of 1∶1 mixture of Hypnorm (Janssen-Cilag Ltd) and Midazolam (Roche) and tissue fixated by intracardial perfusion with 10 ml cold PBS followed by 10 ml of freshly prepared PBS with 4% paraformaldehyde (PFA). A blood sample was obtained by heart puncture prior to perfusion-fixation and EDTA-plasma was isolated by centrifugation (2000 *g*, 30 minutes, 4°C) and stored at −20°C. The tumor located in the fourth breast gland on the left hand side was excised prior to PFA perfusion and stored at −80°C for later RNA purification or extraction for western blotting and zymography. Following PFA perfusion, the corresponding tumor on the right hand side was placed in PFA for paraffin embedding and histological examination. The lungs were removed and placed in PFA for further fixation followed by processing and immunohistochemical staining as described in [41].

### Quantification of metastases

The volume of metastases in lungs was determined by a computer-assisted stereological method on hematoxylin eosin-stained sections of the lungs as described previously in [42].

### Immunohistochemical staining

Immunohistochemical detection of lipocalin-2 and MMP-9 was performed as described in [22]. Antibodies used were goat anti-mouse lipocalin-2 (1∶100, AF1857, R&D Systems) and rabbit anti-mouse MMP-9 (1∶2000, ab38898, Abcam). Immunohistochemical staining of formalin-fixed paraffin-embedded primary tumors for E-cadherin, vimentin, and alpha smooth muscle actin (α-SMA) was performed using the following antibodies: Rabbit anti-mouse E-cadherin (1∶500, ab53033, Abcam), rabbit anti-mouse Vimentin (1∶500, ab92547, Abcam), and rabbit anti-mouse α-SMA (1∶500, ab5694, Abcam). The specimens for this procedure were selected among *PyMT, Lcn2+/+* and *PyMT, Lcn2−/−* mice with large and small total volumes of primary tumors, large, intermediate, and small total volumes of metastases, and among mice with large, intermediate, and small numbers of metastases respectively. In total 16 specimens of primary tumors underwent immunohistochemical staining for E-cadherin, vimentin, and α-SMA. Sections of primary tumors were deparaffinized, hydrated, and antigen retrieval was performed by 10 minutes incubation with proteinase K at 37°C for E-cadherin-staining and 10 minutes of heating at 98°C in citrate buffer, pH 6 for vimentin and α-SMA staining. Endogenous peroxidase activity was blocked with 1% H_2_O_2_. Sections were incubated overnight with primary antibodies diluted in Dako Antibody Diluent (S3022, Dako). Sections were subsequently incubated for 45 minutes with secondary antibody, EnVision^+^-System HRP-Labelled Polymer Anti-rabbit (K4003, Dako), developed with Vector NovaRED (SK-4800, VWR International), counterstained with Mayer's Hematoxylin, and finally dehydrated and mounted. The slides were examined under a BX51 microscope (60x/1.40 PlanApo oil objective) with Olympus DP70 photo system and analySIS software 5.0 (Olympus) or Leica DM 2500 microscope with Leica DFC 425 camera and software.

### Histology

The tumors were diagnosed on coded specimens using the classification of *PyMT* tumors by Lin et al. [43], and employed by Almholt et al. [44]. In short, the four tumor stages are: Hyperplasia, Adenoma, Early Carcinoma, and Late Carcinoma. The patterns of stromal formation were also recorded. Evaluation of sections stained for E-cadherin, vimentin, and α-SMA were evaluated on coded specimens.

### Isolation of murine granulocytes for Western blot

Two FVB/N wild-type mice were euthanized by cervical dislocation and the spleens were removed and homogenized in PBS with 4% fetal calf serum (FCS) with a mortar and a pestle. The homogenate was filtered through a 70 µm nylon mesh (352350, BD Biosciences) and kept on ice. Following centrifugation (300 *g* for 4 minutes, 4°C), the supernatant was removed and the pellet resuspended in Pharm Lyse (BD Biosciences) for lysis of erythrocytes. Lysis was terminated by addition of PBS with 4% FCS and the cells were pelleted by centrifugation (300 *g* for 4 minutes, 4°C) and subsequently resuspended in PBS with 4% FCS. Granulocytic cells were isolated by immunomagnetic sorting using the magnetic-activated cell sorting (MACS) system and Gr-1-Biotin antibody (130-092-332, Miltenyi or 51-01212J, BD Pharminogen) according to the manufacturer's instructions (Miltenyi). Separation was performed on a MACS LS column (Miltenyi).

### Preparation of human samples for Western blot

Granulocytes were isolated from peripheral blood by use of Lymphoprep (Axis-Shield PoC AS) and treated with 5 mM di-isopropyl fluorophosphate (Calbiochem). Granulocytes were resuspended in disruption buffer (100 mM KCL, 1 mM Na_2_ATP, 3.5 mM MgCl_2_, 10 mM PIPES, pH 7.2) with 0.5 mM phenylmethylsulfonylfluoride (PMSF) added. Cells were disrupted by nitrogen cavitation at 600 psi for 5 minutes and collected in 1.5 mM EGTA [45], [46]. The cavitate was centrifuged at 400 *g* for 15 minutes to remove nuclei and unbroken cells. The supernatant (S1), which contains the granules, was analyzed further by Western blot as described below.

### Tumor extraction

Four large tumors from *PyMT, Lcn2+/+* mice and four tumors of corresponding sizes from *PyMT, Lcn2−/−* mice were selected for extraction. The tumors were kept on ice and homogenized with a blender in 5 µl of lysis buffer (150 mM NaCl, 50 mM Trizma base, 0.5% deoxycholic acid, 0.1% SDS, 1% NP-40) per mg weight of tissue. After 15 minutes of incubation on ice the tumor suspensions were centrifuged at 13.500 rpm for 10 minutes at 4°C and the supernatants were collected and stored at −20°C.

### Western blot

The samples were diluted in sample buffer with or without reducing agents and boiled for 5 minutes. Electrophoresis was performed according to standard procedures on a 4–12% Nu Page Bis-Tris gradient gel (Invitrogen) as described in [22]. The antibodies used were goat anti-mouse lipocalin-2 (1∶1000, AF1857, R&D Systems), rabbit anti-mouse MMP-9 (1∶5000, ab38898, Abcam), mouse anti-human lipocalin-2 (1∶1000, [47]) or rabbit anti-human MMP-9 (1∶1000, [46]), followed by HRP-conjugated secondary antibodies: Rabbit anti-goat (1∶1000, P0449, Dako), goat anti-rabbit (1∶1000, P0448, Dako), or rabbit anti-mouse (1∶1000, P0260, Dako). The membranes were developed by chemiluminescence using SuperSignal West Pico Chemiluminescence Substrate (Pierce) according to the manufacturer's instruction and analyzed on a Bio-Rad Chemidoc (Bio-Rad).

### Zymography

Samples in 4% glycerol, 1% SDS, 0.125% bromophenol blue, 125 mM Tris-HCl, pH 6.8 were loaded on precast 10%, 1 mm gelatin zymogram gels (Novex Invitrogen EC61752 BOX) and run at 25 mA, washed in 2.5% Triton X-100, 20 mM Tris-HCl pH 7.8, 5 mM CaCl_2_, 1 µM ZnCl_2,_ incubated with shaking for 1 hour in the same buffer, washed 3 times in H_2_O, incubated overnight at 37°C in 50 ml 1% Triton X-100, 20 mM Tris-HCl pH 7.8, 5 mM CaCl_2_, 1 µM ZnCl_2_, stained for 30 min in 0.5% Coomassie, 10% acetic acid 30% ethanol, and destained in 10% acetic acid, 30% ethanol. For inhibition of metalloproteinase activity, 5 mM EDTA and 2 mM 1,10-phenantroline were added during the overnight incubation [48].

### RNA purification and quantification

Tumors were homogenized and suspended in TRIzol Reagent (Invitrogen) for RNA isolation according to the manufacturer's instruction. mRNA expression was determined by real-time PCR using the TaqMan method as described in [9]. The FAM-labeled probes used were Lcn2: Mm01324470_m1, Mmp9: Mm00600164_g1, Transferrin Receptor (Tfrc): Mm0044941_m1, Megalin (Lrp2): Mm01328171_m1, 24p3R (Slc22a1): Mm00480680_m1, and Bdh2: Mm00459075_m1. For internal normalization we used HEX-labeled Gapdh probe: 4326317E (all from Applied Biosystems).

### Elisa

Quantification of plasma LCN2 was performed by standard ELISA techniques as described in [47] and [30] using the following antibodies: Affinity purified goat anti-lipocalin-2 (1∶500, AF 1857, R&D Systems), biotinylated rabbit anti-lipocalin-2 (1∶200, [30]), and horseradish peroxidase Avidin D (1∶3000, 18–4100, eBioscience).

### Statistics

Statistical analyses were performed using GraphPad Prism 5 (GraphPad Software). To meet the requirements of parametric tests, we log10 transformed tumor volumes. Significance was set at p<0.05.

## Results

**Figure 1:**
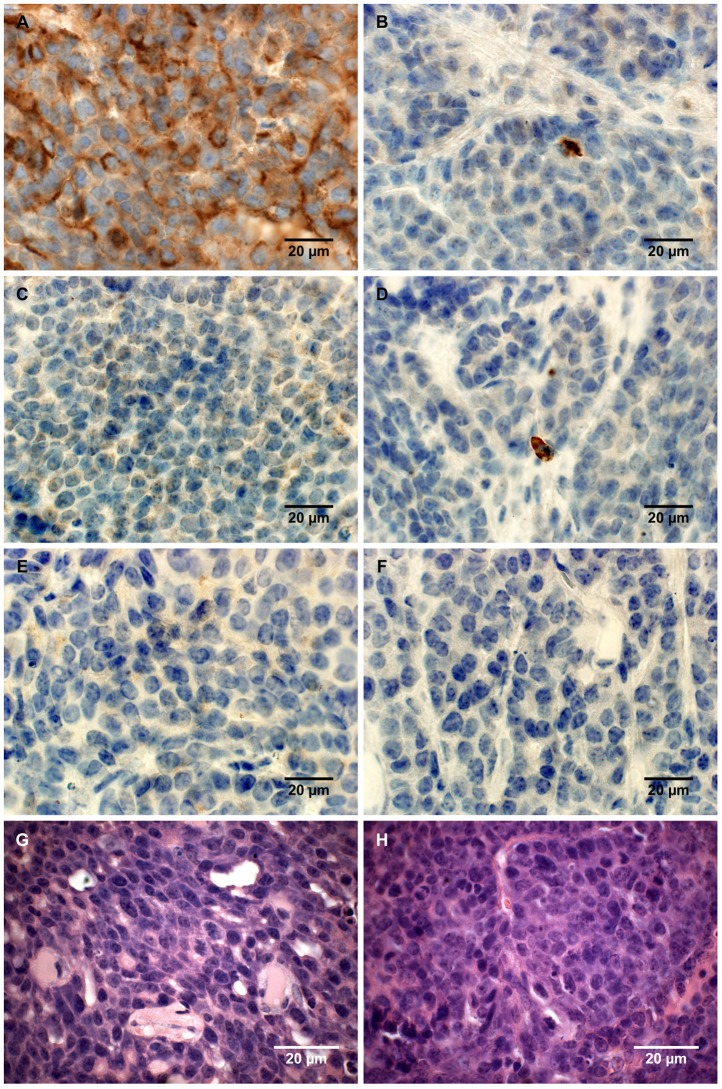
Histological characterization of mammary tumors. A, C, E, immunohistochemical staining for lipocalin-2 in mammary tumor in (A) *PyMT, Lcn2*+/+ mouse, (C) *PyMT, Lcn2*−/−, and (E) *PyMT, Lcn2*+/+ mouse as negative control, where no primary antibody was added. B, D, F, immunohistochemical staining for MMP-9 in mammary tumor in (B) *PyMT, Lcn2*+/+ mouse, (D) *PyMT, Lcn2*−/−, and (F) *PyMT, Lcn2*+/+ mouse as negative control, where no primary antibody was added. Original magnification x600. G, H+E staining of tumor from a *PyMT, Lcn2*+/+ mouse representing largest metastasis volume. F, H+E staining of tumor from *PyMT, Lcn2*−/− mouse representing largest metastasis volume. In both G and F strongly atypical tumor cells with numerous mitoses are seen, and surrounded by slender strands of collagen tissue. Original magnification x630. Abbreviations: *PyMT: MMTV-PyMT*.

**Figure 2:**
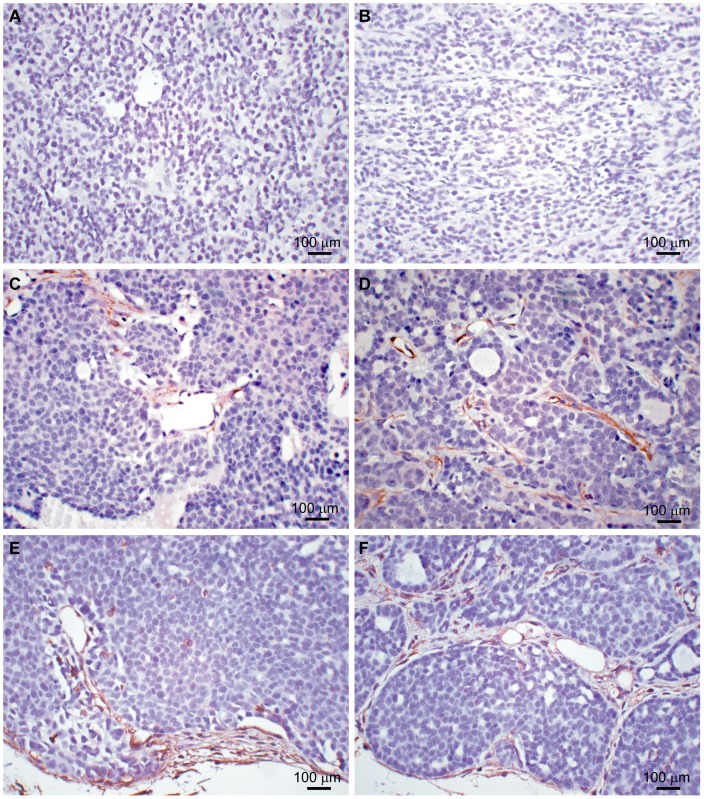
Representative immunohistochemical stainings of mammary tumors. A, staining for E-cadherin in a *PyMT, Lcn2+/+* mouse. B, staining for E-cadherin in a *PyMT, Lcn2−/−* mouse. C, staining for α-SMA in a *PyMT, Lcn2+/+* mouse. D, staining for α-SMA in a *PyMT, Lcn2−/−* mouse. E, staining for vimentin in a *PyMT, Lcn2+/+* mouse. F, staining for vimentin in a *PyMT, Lcn2−/−* mouse. Original magnification x400. Abbreviations: *PyMT: MMTV-PyMT*. α-SMA: Alpha smooth muscle actin.

We chose the *MMTV-PyMT*-model on a FVB/N background for the study of spontaneous breast cancer development as tumor development is very uniform and predictable with time, thus offering minimal variation in tumor development and progression [49], [50]. The breeding strategy is shown in Figure S1. The progression of mammary tumors was quantified as described in Materials and Methods and the mice were sacrificed at week 13. Figure 1 (A, C, E) shows the expression of lipocalin-2 in tumors of *MMTV-PyMT* wild-type mice and *MMTV-PyMT Lcn2* knock-out mice (from now on referred to as *PyMT*-mice). A uniform strong immunohistochemical staining for lipocalin-2 was observed in the tumor cells of wild-type mice whereas no labeling was observed in the knock-out mice. MMP-9 was observed in the few neutrophils present and no difference was observed between wild-type and *Lcn2* knock-out mice (Figure 1 B, D, F). Histological examination showed that all tumors except one were Late Carcinomas. The only Early Carcinoma was from the group with lowest numbers of metastases, indicating low malignant potential. No obvious differences in the stroma were observed between the two groups. In Late Carcinomas, fibrous strands of collagen tissue were seen between tumor nodules, and tumors were otherwise surrounded by loose connective tissue or fatty tissue. Focal squamous differentiation was seen in one Late Carcinoma, a feature which is not considered to alter the malignant potential. In the other Late Carcinomas, smaller and larger cystic necroses or partly cystic glands were seen. Hence, no difference in indices of malignancy was observed between wild-type and knock-out mice as determined by histological examination (Figure 1 G, H and table 1). Moreover, we performed immunohistochemical staining for the luminal marker E-cadherin, the myoepithelial marker alpha smooth muscle actin (α-SMA), and the mesenchymal marker vimentin. E-cadherin was negative in all tumors (Figure 2 A, B) but clearly positive in adjacent normal mammary glands and epidermis (Figure S2 A). α-SMA was also negative in all tumors, but some positivity was seen in tumor capsule and vessel wall (Figure 2 C, D). Normal mammary glands with myoepithelial cells and vessel walls stained positive for α-SMA as expected (Figure S2 B). Vimentin stained positive in tumor capsule and in small connective tissue strands between tumor nodules, but the core of the tumor nodules was negative (Figure 2 E, F). This is conformable with the observations performed on H+E-stained sections where very little stroma is seen in tumors. Overall we saw no differences in staining for E-cadherin, vimentin and α-SMA between tumors from *PyMT, Lcn2+/+* and *PyMT, Lcn2−/−* mice.

**Table 1:** Histological diagnoses of H+E-stainings of the breast tumors.

Selection criteria	Histology, wild type	Histology, knock out
Largest total volume of primary tumors	LC	LC
Smallest total volume of primary tumors	LC	LC
Largest number of metastases	LC	LC
Smallest number of metastases	LC	EC
Largest total volume of metastases	LC	LC
Smallest total volume of metastases	LC	LC

Tumors diagnosed were selected by the above listed criteria.

LC =  Late Carcinoma EC =  Early Carcinoma.

We next assessed the influence of lipocalin-2 on the following parameters of tumor growth and metastases: Age at appearance of palpable tumors, volume of primary tumors at sacrifice, number of mammary glands involved, total volume of metastases, number of metastases, and volume of primary tumors versus volume of metastases.

**Figure 3:**
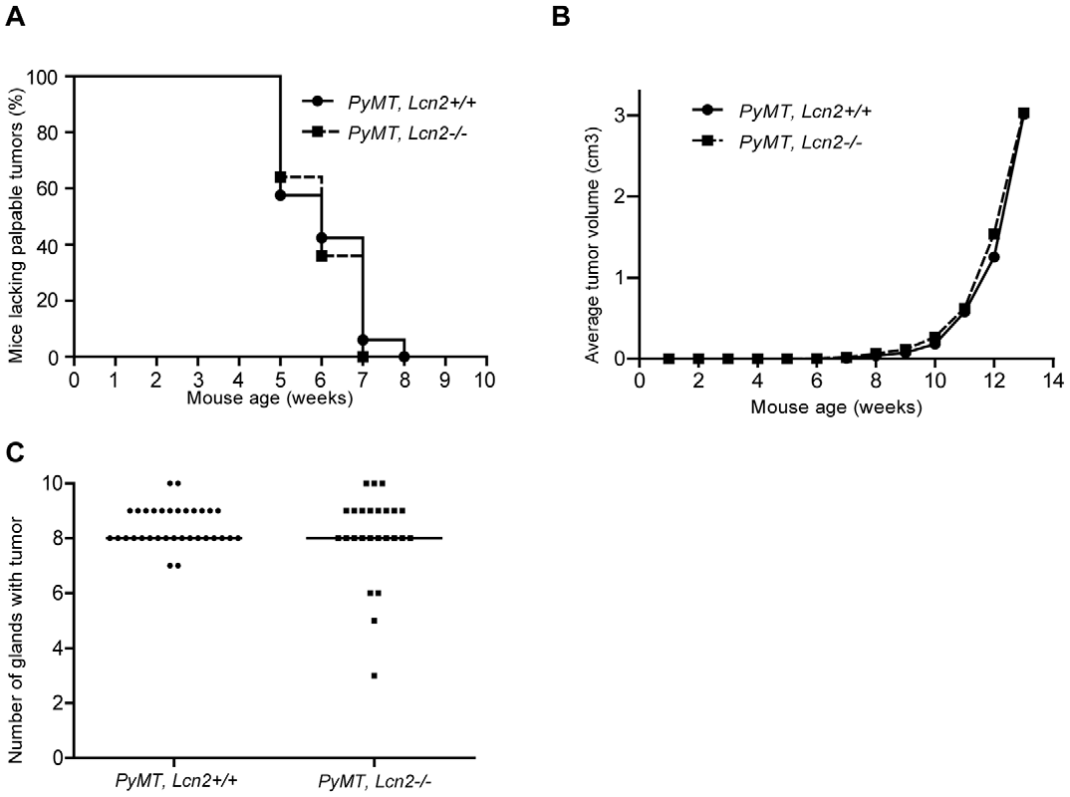
Growth of primary tumors in *PyMT*, *Lcn2*+/+ and *PyMT*, *Lcn2*−/− mice. A, *Lcn2*-deficiency has no effect on tumor onset. Data shown are percentage of mice without palpable mammary tumors on the indicated weeks visualized by a Kaplan-Meier plot. Curves did not differ statistical significantly, log-rank test, p = 0.57 (n = 33 for *PyMT, Lcn2*+/+ and n = 25 for *PyMT, Lcn2*−/−). B, average of total volume of mammary tumors versus age. No statistical significant difference between *PyMT, Lcn2*+/+ and *PyMT, Lcn2*−/− mice at week 13, t-test after logarithmic transformation, p = 0.21 (n = 29 for *PyMT, Lcn2*+/+ and n = 19 for *PyMT, Lcn2*−/−). C, numbers of tumor positive glands per mouse as determined by palpation at week 10 where the first mouse out of all the mice presents with all mammary glands tumor positive. No significant difference in tumor positive mammary glands, Mann-Whitney *U*-test, P = 0.85 (n = 33 for *PyMT, Lcn2*+/+ and n = 25 for *PyMT, Lcn2*−/−). Horizontal bars indicate medians. Abbreviations: *PyMT: MMTV-PyMT*.

Tumor onset occurred early in this FVB/N background mouse. By week 5, 40% of both wild-type (14 of 33) and *Lcn2* knock-out mice (9 of 25) developed palpable tumors and by week 8 all mice (n = 58) had palpable tumors. No statistical significant difference was observed between the two groups of mice (Figure 3 A). The average total tumor volume, as determined by a caliper, increased exponentially from week 8 until sacrifice and no difference was observed between the two groups (Figure 3 B). The same holds true for the mammary gland pair with the least aggressive growth rate (pair number two) and the pair with the most aggressive growth rate (pair number one), see Figure S3. At sacrifice, the vast majority of mice had tumors in 8 or more of the 10 mammary glands, and again no statistical significant difference was observed between the two groups (Figure 3 C). The uniform development of mammary tumors in this mouse model is quite striking.

**Figure 4:**
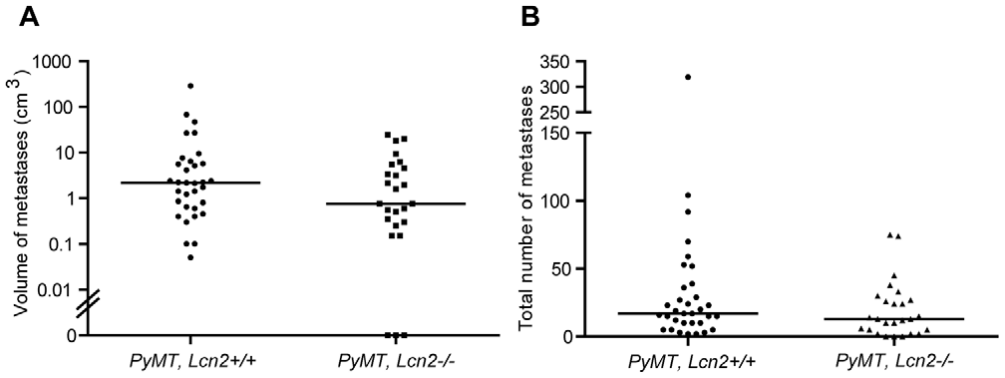
Growth of metastases in *PyMT*, *Lcn2*+/+ and *PyMT*, *Lcn2*−/− mice. Lipocalin-2-deficiency has no statistical significant effect on total volume (t-test, p = 0.09) or number (t-test, p = 0.09) of lung metastases derived from primary mammary tumors (n = 33 for *PyMT, Lcn2*+/+ and n = 26 for *PyMT, Lcn2*−/−). A, volumes of lung metastases in *PyMT*, *Lcn2*+/+ and *PyMT, Lcn2*−/− mice. Dots at 0 indicate mice without metastases. B, total numbers of lung metastases in *PyMT, Lcn2*+/+ and *PyMT*, *Lcn2*−/− mice. Horizontal bars indicate medians. Abbreviations: *PyMT: MMTV-PyMT*.

The effect of lipocalin-2 on number and total volume of metastases was determined as described in Material and Methods. The range, both in number of metastases and in their cumulated volume, is very wide ranging from three mice without metastases in the *Lcn2* knock-out group to more than 300 metastases in one wild-type mouse. No statistical significant difference was observed between the two groups with respect to either number of metastases or total volume of metastases (Figure 4). A significant correlation was observed between the volume of primary tumors and the volume of metastases (p<0.01). The same relation was found in wild-type and *Lcn2* knock-out mice (data not shown).

**Figure 5:**
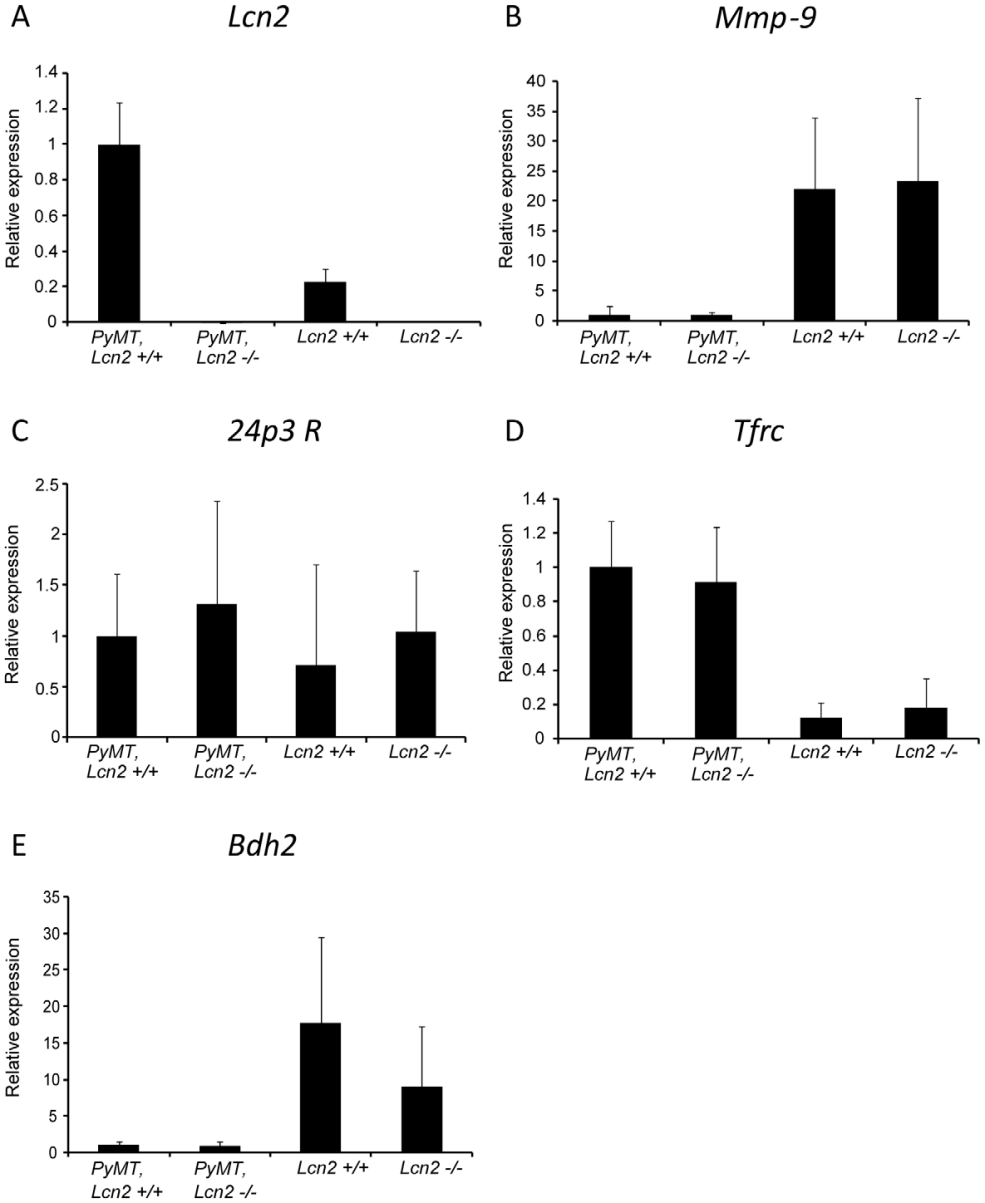
Quantitative real-time PCR analysis of mRNA levels in mammary tumors/glands. Expression levels of each marker are shown relative to the value found in tumors of *PyMT, Lcn2*+/+ mice, which is given the value 1. The cycle threshold (Ct) value of the *PyMT, Lcn2*+/+ mouse with the highest expression of each marker is: Lcn2 (Ct = 20), Mmp-9 (Ct = 34), 24p3R (Ct = 28), Tfrc (Ct = 27), and Bdh2 (Ct = 35). Data for the *PyMT, Lcn2*+/+ and *PyMT, Lcn2*−/− mice are the mean expression in tumors of nine mice each (each measured in triplicate). Data for the *Lcn2*+/+ and *Lcn2*−/− mice represent the mean expression in mammary glands of two mice each (each measured in triplicate). The vertical error bars represent the standard deviations. Abbreviations: *PyMT: MMTV-PyMT*.

In order to explore any relation of tumor aggressiveness and lipocalin-2 expression to pathways which are relevant for mediating effects of lipocalin-2 or for compensating the lack of lipocalin-2, we systematically investigated the mRNA levels of such relevant proteins in samples from nine wild-type and nine *Lcn2* knock-out mice. The size of tumor in the fourth left mammary gland was determined in each mouse and three sample sets consisting of three animals having small tumors, three with intermediate size tumors, and three with large tumors were selected, and mRNA extracted for quantitative real-time PCR. Expression of mRNA for Lcn2, Mmp9, Bdh2, 24p3R, megalin (another receptor mediating cellular uptake of lipocalin-2 [51]), and transferrin receptor (Tfrc) were determined in each tumor and reported as mean of all tumors/tissue samples in each of the four groups of mice (Figure 5). The values for each of the three sample sets for the two *PyMT*-positive mouse populations are given in Figure S4. Figure 5 A shows that Lcn2 mRNA was upregulated 5 fold in tumors of *PyMT, Lcn2*+/+ mice compared to normal mammary tissue of wild-type mice, whereas Mmp9 mRNA was virtually absent from tumors, but present in normal mammary tissue with no difference between the two groups of mice (Figure 5 B). No signal was observed for megalin (data not shown) whereas expression of the other lipocalin-2 receptor 24p3R was detected but showed no difference between *PyMT, Lcn2+/+* and *PyMT, Lcn2−/−* mice or between *PyMT*-positive and *PyMT*-negative mice (Figure 5 C). We speculated that a compensatory increase of the transferrin receptor might take place in the absence of lipocalin-2. The transferrin receptor was over-expressed in tumors compared to normal tissue, but with no significant difference between *PyMT, Lcn2+/+* and *PyMT, Lcn2−/−* mice (Figure 5 D). mRNA for the enzyme BDH2, which produces 2,5-DHBA, the recently identified mammalian siderophore ligand for lipocalin-2, was barely detected in normal mammary tissue as estimated by the late appearance of BDH2 in our real-time PCR, and was virtually absent in tumors with no difference between the two groups of mice (Figure 5 E).

**Figure 6:**
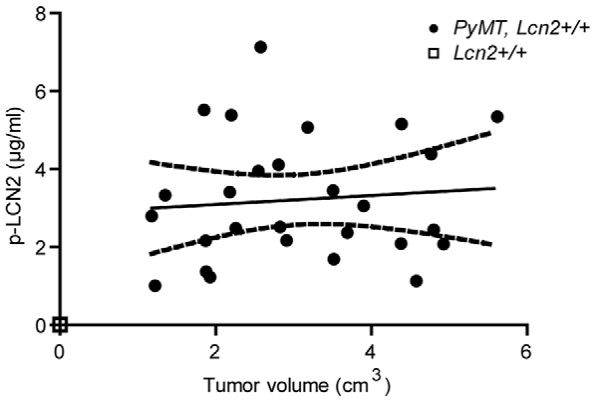
Plasma-lipocalin-2 (p-LCN2) analyzed by ELISA. There is no correlation determined by linear regression between the total volume of mammary tumors at week 13 and p-LCN2 in *PyMT*, *Lcn2*+/+ mice, p = 0.68 (n = 27). *PyMT, Lcn2*+/+ mice are indicated by dots and *Lcn2*+/+ mice are indicated by a square. Solid line indicates best fitted line and dashed lines indicate 95% confidence bands. Abbreviations: *PyMT: MMTV-PyMT*.

In contrast to man [30], lipocalin-2 is generated by the mouse liver as an acute phase protein [52]. We therefore expected the level of lipocalin-2 in plasma to increase with increasing tumor burden as a result of secretion both from the tumors and from the liver. We did not, however, observe any statistical significant increase with tumor burden (Figure 6).

**Figure 7:**
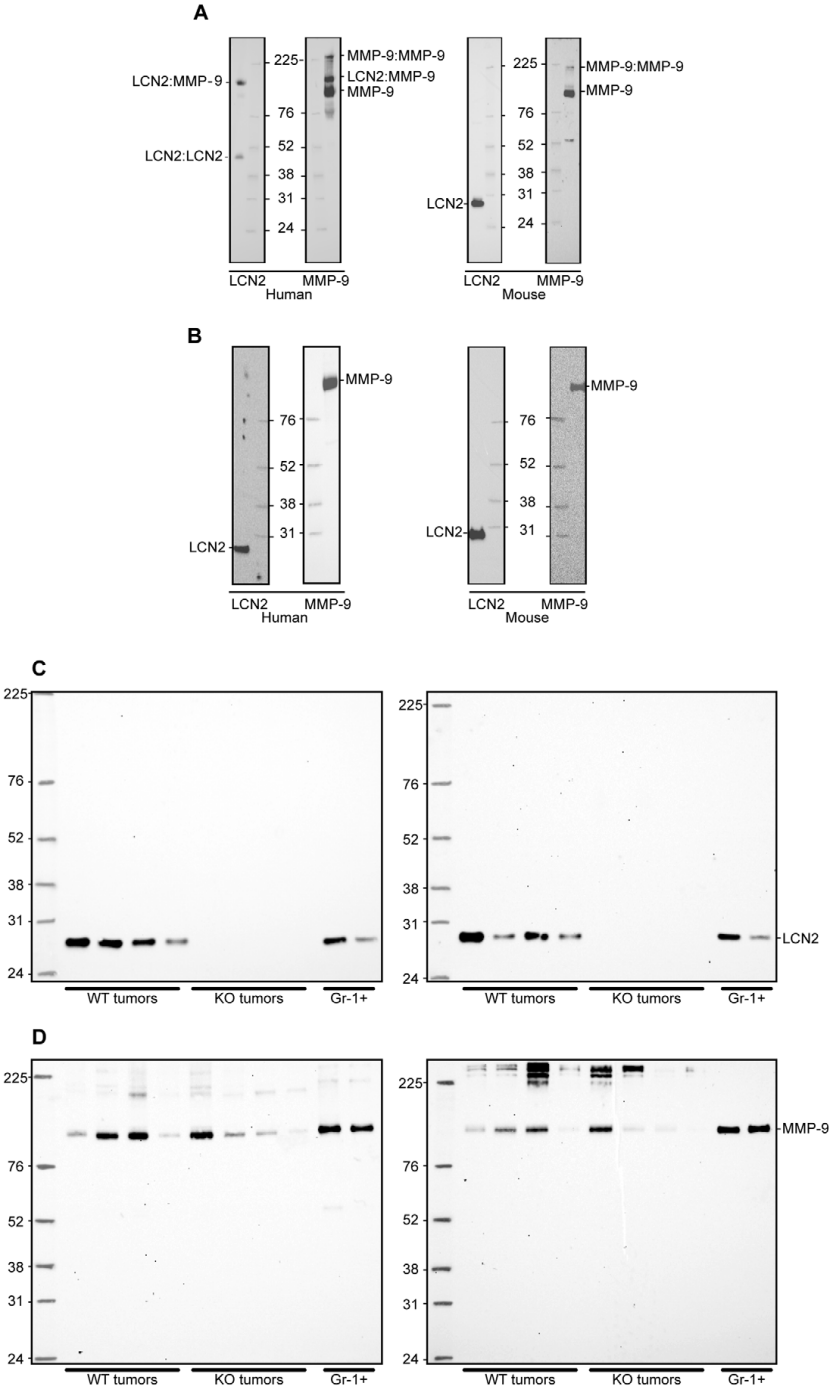
Western blot of human granulocytes from peripheral blood, mouse granulocytes from the spleen, and tumor extracts. Lipocalin-2 (LCN2) is unable to form a heterodimer with MMP-9 in mouse. There is no difference in the post translational modification of LCN2 in tumors compared to myeloid cells in mouse. A, samples analyzed under non-reducing conditions. In humans the LCN2:LCN2 and the MMP-9:MMP-9 homodimers and the LCN2:MMP-9 heterodimer are identified. In addition the MMP-9 monomer is seen. In contrast no heterodimer between LCN2 and MMP-9 is seen in the mouse, only the LCN2 monomer and both the monomeric and dimeric forms of MMP-9 are seen. B, samples analyzed under reducing conditions. The LCN2:MMP-9 heterodimer as well as the LCN2:LCN2 and MMP9:MMP9 homodimers are no longer identifiable after breakage of disulfide bindings. Merely the LCN2 monomer and the MMP-9 monomer are identified in both humans and mouse. C, left panel, samples analyzed under non-reducing conditions. Right panel, reducing conditions. Only the LCN2 monomer and no heterodimer is identified under both conditions. D, left panel, samples analyzed under non-reducing conditions. The MMP-9 monomer is seen, and in some of the tumors even the homodimer is identified. Right panel, reducing conditions. The MMP-9 monomer is seen along with high molecular weight (HMW) bands of unknown origin. The HMW bands are seen in both *PyMT, Lcn2+/+* and *PyMT, Lcn2−/−* mice and therefore cannot be the lipocalin-2:MMP-9 heterodimer. All blots are shown in full-length size. Samples are loaded in the same order in C and D as in Figure 8. Abbreviations: WT and KO tumors: Tumors from *PyMT, Lcn2+/+* or *PyMT, Lcn2−/−* mice respectively. Gr-1+: Gr-1 positive cells purified from the spleen of two FVB/N mice.

Both previous reports on spontaneous transgenic breast cancer in a lipocalin-2 knock-out or wild-type background [38], [39] have identified a high-molecular weight form of MMP-9. In one study no evidence for the formation of a lipocalin-2:MMP9 complex was found [38] whereas in the second study [39] this high-molecular weight form of MMP-9 was interpreted as an association between MMP-9 and lipocalin-2 and presented as a possible explanation for the enhanced aggressiveness of the tumors expressing lipocalin-2 as lipocalin-2 was suggested to prevent degradation of MMP-9 [39]. The latter interpretation is surprising as the formation of a heterodimer between lipocalin-2 and MMP-9 depends on the availability of a free cysteine in both proteins. The human lipocalin-2, NGAL, contains three cysteines, two of which (Cys 76 and 175) are engaged in an intramolecular disulfide loop, and the third (Cys 87) available for formation of homodimers or heterodimers, which may involve 92 kD MMP-9 [18]. The mouse orthologue, however, contains only the two cysteines engaged in intramolecular disulfide loops and the mouse lipocalin-2 is consequently unable to form both homodimers and heterodimers. This was ascertained by western blotting of lipocalin-2 and MMP-9 in tumors and myeloid cells under reducing and non-reducing conditions from wild type and Lcn2 knock-out mice and compared with human myeloid cells (Figure 7 A–D). The same molecular weight (MW) form corresponding to LCN2 monomer is observed in tumors and myeloid cells, indicating that there is no difference in post translational modification of LCN2 in tumors and myeloid cells in mice (Figure 7 C). Only monomeric LCN2 is observed in tumors and myeloid cells under non-reducing conditions and no band corresponding to the LCN2:MMP-9 heterodimer was observed. In Figure 7 D immunoreactive bands with very high MW forms are observed in tumors from both wild type and Lcn2 knock-out. The nature of these is unknown but these cannot represent complexes between MMP-9 and lipocalin-2 as the same bands are observed for both wild type and lipocalin-2 knock-out mice.

**Figure 8:**
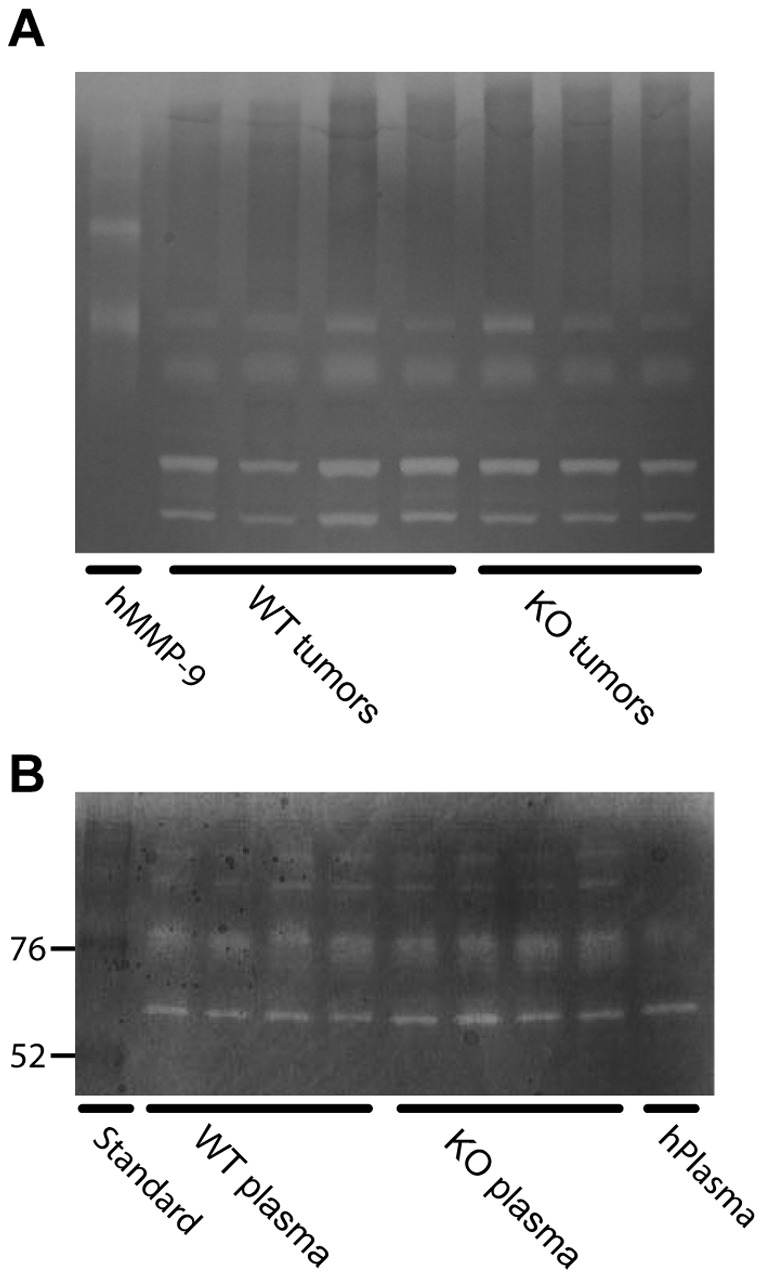
Gelatin zymography of MMP-9 activity. No difference in gelatinolytic activity is observed between *PyMT, Lcn2+/+* and *PyMT, Lcn2−/−* mice, either in tumors or in plasma. A, zymography of tumors from *PyMT, Lcn2*+/+ (WT) and *PyMT, Lcn2*−/− (KO) mice with purified human MMP-9 (hMMP-9) as standard [2]. B, zymography of plasma from WT and KO mice along with human plasma and a molecular weight standard. Tumor and plasma samples from mice are loaded in the same order as in Figure 7 C and D.

In addition, we performed zymography of mouse plasma and tumor extracts from wild type and Lcn2 knock-out mice (Figure 8 A and B). We observed several bands with gelatinolytic activity both in plasma and in tumor extracts, but with no difference in the patterns between wild type and Lcn2 knock-out. No activity with a MW higher than the MW of monomeric human MMP-9 was observed in tumors. All gelatinolytic activity was inhibited by EDTA and phenantroline (data not shown).

## Discussion

Our results show that lipocalin-2 is dispensable for tumor development judged by any of the parameters used for determination of the clinical aggressiveness of cancers, such as differentiation grade, time to cancer development, number of mammary glands involved, size of primary tumors, and number and size of metastases in this mouse model of breast cancer induced by the mouse mammary tumor virus-polyoma middle T antigen.

This is in contrast to two recent reports using much the same approach as here to study the role of lipocalin-2 in breast cancer development. The first report, where tumor development was driven by the *ErbB2* oncogene [39], showed both faster onset of tumor genesis, higher primary tumor numbers, larger volume of primary tumors, as well as a higher number of metastases and larger volume of metastases in lipocalin-2 expressing mice. The second report, where the tumors were driven by the polyoma-middle T antigen as in our study, showed no effect of lipocalin-2 expression on number of metastases, but did show an effect on number of primary tumors, the volume of primary tumors, and on the time of onset of tumor genesis [38]. The mice used for examining the effect of *ErbB2*-induced mammary tumors was obtained by breeding Lcn2−/− (C57BL/6) mice with MMTV-ErbB2 (FVB/N) mice. The mixed and non-homogeneous genetic background of the offspring from the cross between the two mouse strains (C57BL/6 and FVB/N) in combination with the smaller sample sizes analyzed in the *ErbB2*-study might explain some of the differences between the finding and the data presented here. The other study examining *PyMT*-induced mammary tumors was performed in both C57BL/6 and 129/Ola mice (both backcrossed for at least 10 generations), while we used mice on a FVB/N background backcrossed for 8 generations. It is possible that this may have had an effect on the outcome. The FVB/N strain has been demonstrated to be more susceptible than C57BL/6 with respect to tumor genesis in the *PyMT* model [49], [53], [54]. The FVB/N background has therefore been used by us for several studies of mammary tumor growth and metastases because it offers a very uniform and predictable mammary tumor development by the tumor virus-polyoma middle T antigen [55], thus optimizing the ability to test the effect of agents that interfere with tumor development, in particular such that inhibit tumor growth and metastases [44], [56]. Finally, one could envision that the specific knock-out mouse model used might influence the results. Berger et al. used a knock-out mouse constructed by themselves [21] while the Lcn2 knock-out mouse used in the Leng study and by us is developed by Aderem and Akira [20].

There are multiple possible reasons for our observation of the lack of effect of lipocalin-2 in the face of several other studies indicating the contrary. It is possible that the growth potential of the tumor cells and their propensity for metastasis in our model is so strong that it overrides a modulating effect of lipocalin-2. Tumor genesis occurred very early in our study as all mice had palpable tumors by day 56 versus only 10% mice with palpable tumors on day 56 in the *PyMT* model by Berger et al [38]. Tumors appeared even later in the *ErbB2* mouse (100% tumor free day 160) [39]. Thus, it seems that the largest effect of lipocalin-2 on tumor development and aggressiveness is observed in the mouse model with the slowest onset of tumor genesis, the *ErbB2* mouse. A contributing factor to the difference could be that the *ErbB2* and *PyMT* oncogenes work through different signaling pathways [39], [50]. We do, however, consider it unlikely that the effect of lipocalin-2 diminishes with the aggressiveness of the tumors if lipocalin-2 promotes tumor growth by offering iron for cell division, as such effect would not be overruled by a strong intrinsic drive for cell division and metastasis. In fact the contrary would be expected, i.e. a high sensitivity towards deprivation of lipocalin-2. We also believe that the high number of animals in each arm of our cohort and the relatively minor variation in the two groups would allow even minor effects of lipocalin-2 to be picked up.

Another explanation would be that the expression of lipocalin-2 in mammary tumors is significantly lower in our study than the expression in the mammary tumors in the two other reports. While we do not have any data to allow a direct comparison, the expression of lipocalin-2 was certainly pronounced in the mammary tumors in our study as seen in Figure 1 and as indicated by the quantitative mRNA data (Figure 5). Yet another explanation could be that lipocalin-2 cannot be utilized properly by the tumor cells in our mouse system. While the lipocalin-2 receptor megalin was absent, 24p3R mRNA was expressed in the tumors allowing for a possible uptake of lipocalin-2 in these cells. We did, however, only obtain a very weak signal for mRNA for enzyme BDH2 that catalyzes the synthesis of 2,5-DHBA, which renders it unlikely that this mammalian siderophore is involved in iron homeostasis of these cells. This could explain why the lack of lipocalin-2 production in our knock-out mice did not have any effect on mammary cancer development in the FVB/N mouse strain. Whether the expression of the lipocalin-2 receptors and BDH2 is similar in the C57BL/6 and 129/Ola strains used in the other studies is not known, as no report of this was given in the studies.

It is well established that the different genetic backgrounds of the FVB/N and C57BL/6 mouse strains can affect the outcome of a genetic ablation on cancer development as exemplified in the *Mmp-9* [54] and *iNOS* [53] knock-out models. This may also be the case with lipocalin-2 indicating that the effect of *Lcn2* on cancer development is dependent on the genetic setting of the individual organism.

Another mechanism by which lipocalin-2 can convey a metastatic potential to tumor cells has been proposed to relate to the possible activating or stabilizing effect of lipocalin-2 on MMP-9 [34], [57]. However, our data do not support such a mechanism. First, we did not observe any enhanced expression of MMP-9 in the lipocalin-2 expressing mice. Second, we show that mouse lipocalin-2, in contrast to the human orthologue, lacks the ability to form a covalent bond with MMP-9 and hence does not have the potential to mediate MMP-9 activation or inhibited degradation of MMP-9 as has been inferred. This is also in agreement with our findings on zymography. Our investigation therefore leads us to conclude that the role of lipocalin-2 in breast cancer progression is questionable.

## Supporting Information

Figure S1 **Breeding strategy used for generating the mice used in the experiments.** Congenic heterozygous male FVB/N-*MMTV-PyMT* mice were mated with female FVB/N-*Lcn2*+/− mice back-crossed to the FVB/N strain for 8 generations (N8). Their male FVB/N-*PyMT, Lcn2*+/− offspring (F1) were mated with FVB/N-*Lcn2*+/− (N9) females to generate the FVB/N-*PyMT, Lcn2*+/+; FVB/N-*PyMT, Lcn2*−/−; FVB/N *Lcn2*+/+, and FVB/N, *Lcn2*−/− mice used throughout the study. Abbreviations: *PyMT: MMTV-PyMT*.
(TIF)

Figure S2 **Positive control stainings for E-cadherin and α-SMA.** A, E-cadherin staining of the dermis and epidermis of a *PyMT, Lcn2+/+* mouse as positive control to Figure 2 A, B. Original magnification x400. B, α-SMA staining of a primary tumor and adjacent vessels of a *PyMT, Lcn2+/+* mouse as positive control to Figure 2 C, D. Original magnification x200. Abbreviations: *PyMT: MMTV-PyMT*.
(TIF)

Figure S3 **Tumor growth of the fastest and the slowest growing mammary gland pairs in** ***PyMT, Lcn2+/+*** **and** ***PyMT, Lcn2−/−*** **mice.** A, average of tumor volume in gland pair number one versus mouse age. B, average of tumor volume in gland pair number two versus mouse age. No statistical significant difference between *PyMT, Lcn2+/+* and *PyMT, Lcn2−/−* mice at week 13, t-test after logarithmic transformation, p = 0.60 for gland pair number one and p = 0.51 for gland pair number two (n = 29 for *PyMT, Lcn2+/+* and n = 20 for *PyMT, Lcn2−/−*).
(TIF)

Figure S4 **Quantitative real-time PCR analysis of mRNA levels in mammary tumors/glands.** A, Lcn2. B, Mmp9. C, 24p3R. D, Tfrc. E, Bdh2. The vertical error bars represent standard deviations. The mean expressions are shown in three *PyMT, Lcn2+/+* mice with small tumors (1), three with intermediate size tumors (2) and three with large tumors (3), and the mean expressions are shown in three *PyMT, Lcn2-/-* mice with small tumors (4), three with intermediate size tumors (5) and three with large tumors (6) located to the fourth mammary gland on the left hand side. The *PyMT* negative mice were selected randomly and number (7) and (8) represent two mice each. Expression levels of each marker are shown relative to the value found in small tumors of *PyMT, Lcn2+/+* mice, which is given the value 1. Tumor volumes: 1) 0.07 cm^3^, 0.15 cm^3^, 0.17 cm^3^ 2) 0.47 cm^3^, 0.55 cm^3^, 0.68 cm^3^ 3) 0.82 cm^3^, 0.98 cm^3^, 1.50 cm^3^, 4) 0.02 cm^3^, 0.08 cm^3^, 0.09 cm^3^ 5) 0.50 cm^3^, 0.54 cm^3^, 0.63 cm^3^ 6) 0.73 cm^3^, 0.98 cm^3^, 1.33 cm^3^ Abbreviations: *PyMT: MMTV-PyMT*.
(TIF)
