# Supplementary figures and images for: No Effect of NGAL/lipocalin-2 on Aggressiveness of Cancer in the MMTV-PyMT/FVB/N Mouse Model for Breast Cancer
Source: PLoS One. 2012 Jun 21;7(6):e39646. doi: 10.1371/journal.pone.0039646 (PMC3380857; doi:10.1371/journal.pone.0039646)

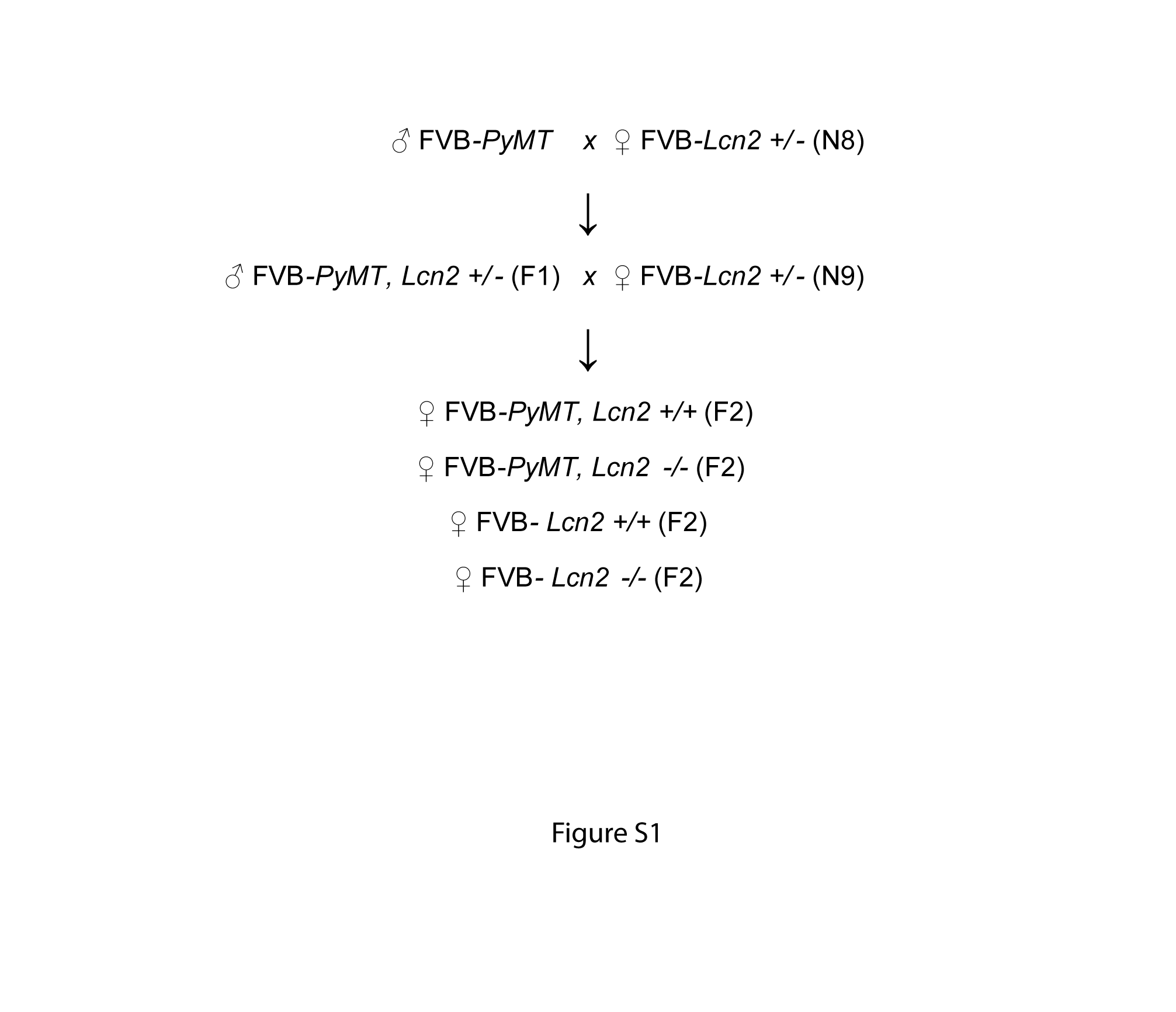

Supplement: Figure S1 — Breeding strategy used for generating the mice used in the experiments. Congenic heterozygous male FVB/N-MMTV-PyMT mice were mated with female FVB/N-Lcn2+/− mice back-crossed to the FVB/N strain for 8 generations (N8). Their male FVB/N-PyMT, Lcn2+/− offspring (F1) were mated with FVB/N-Lcn2+/− (N9) females to generate the FVB/N-PyMT, Lcn2+/+; FVB/N-PyMT, Lcn2−/−; FVB/N Lcn2+/+, and FVB/N, Lcn2−/− mice used throughout the study. Abbreviations: PyMT: MMTV-PyMT. (TIF) [file pone.0039646.s001.tif]

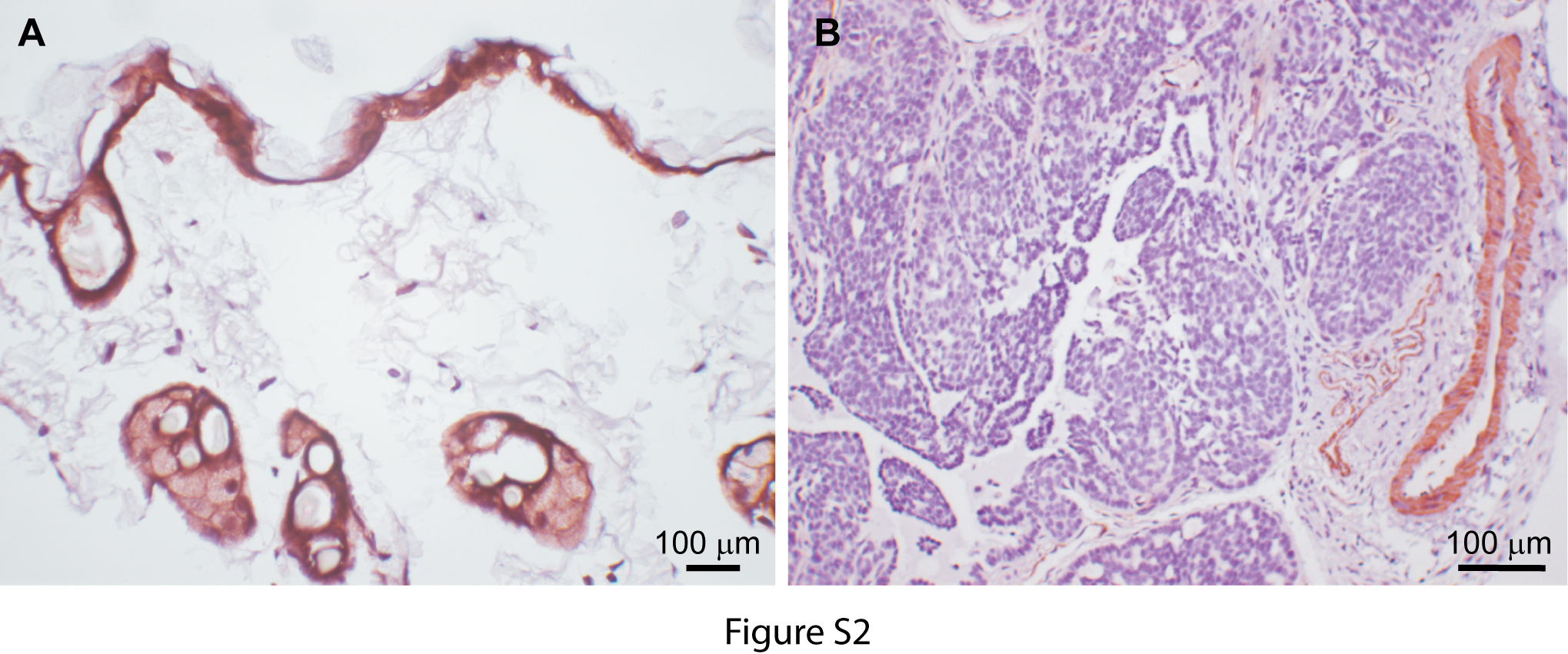

Supplement: Figure S2 — Positive control stainings for E-cadherin and α-SMA. A, E-cadherin staining of the dermis and epidermis of a PyMT, Lcn2+/+ mouse as positive control to Figure 2 A, B. Original magnification x400. B, α-SMA staining of a primary tumor and adjacent vessels of a PyMT, Lcn2+/+ mouse as positive control to Figure 2 C, D. Original magnification x200. Abbreviations: PyMT: MMTV-PyMT. (TIF) [file pone.0039646.s002.tif]

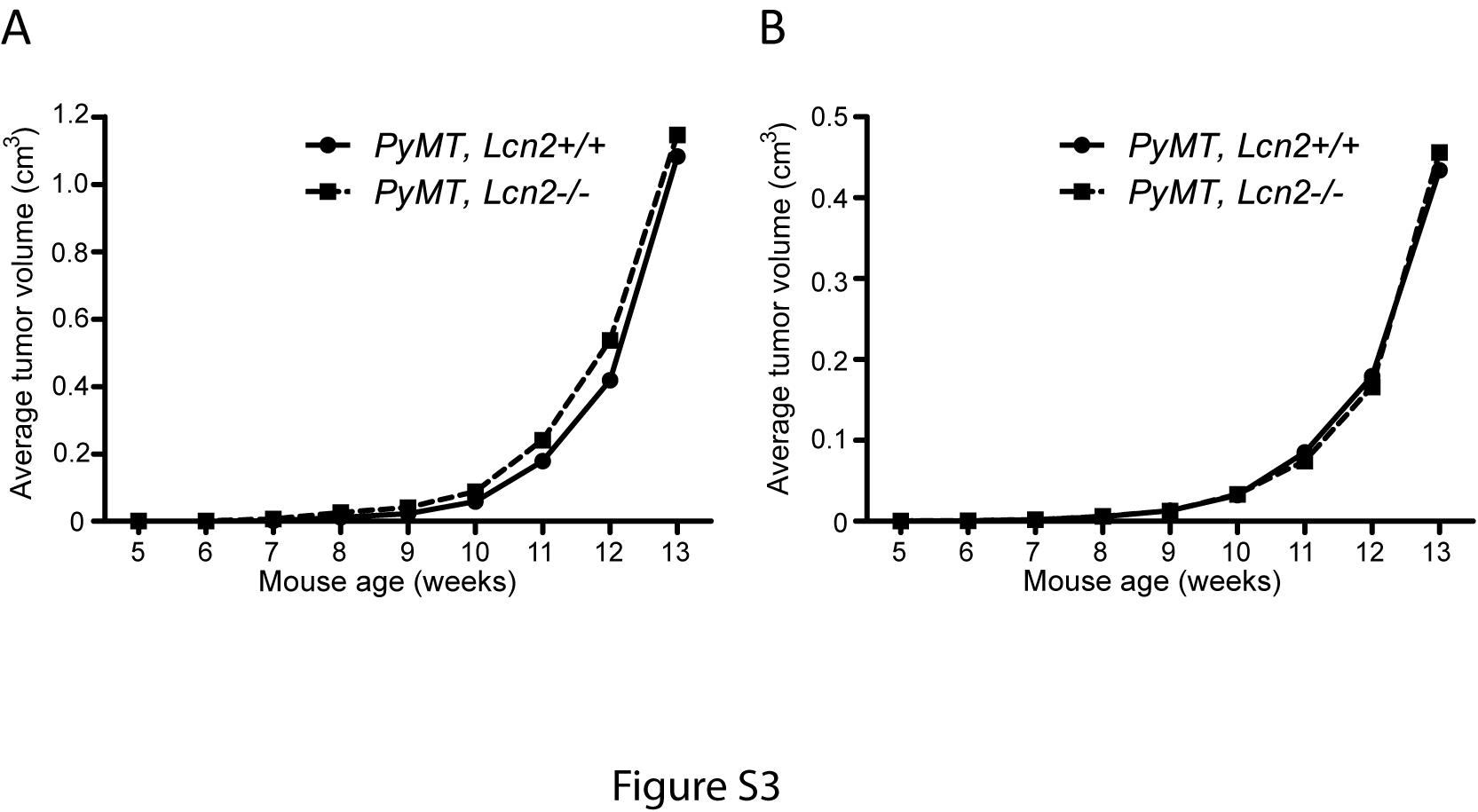

Supplement: Figure S3 — Tumor growth of the fastest and the slowest growing mammary gland pairs in PyMT, Lcn2+/+ and PyMT, Lcn2−/− mice. A, average of tumor volume in gland pair number one versus mouse age. B, average of tumor volume in gland pair number two versus mouse age. No statistical significant difference between PyMT, Lcn2+/+ and PyMT, Lcn2−/− mice at week 13, t-test after logarithmic transformation, p = 0.60 for gland pair number one and p = 0.51 for gland pair number two (n = 29 for PyMT, Lcn2+/+ and n = 20 for PyMT, Lcn2−/−). (TIF) [file pone.0039646.s003.tif]

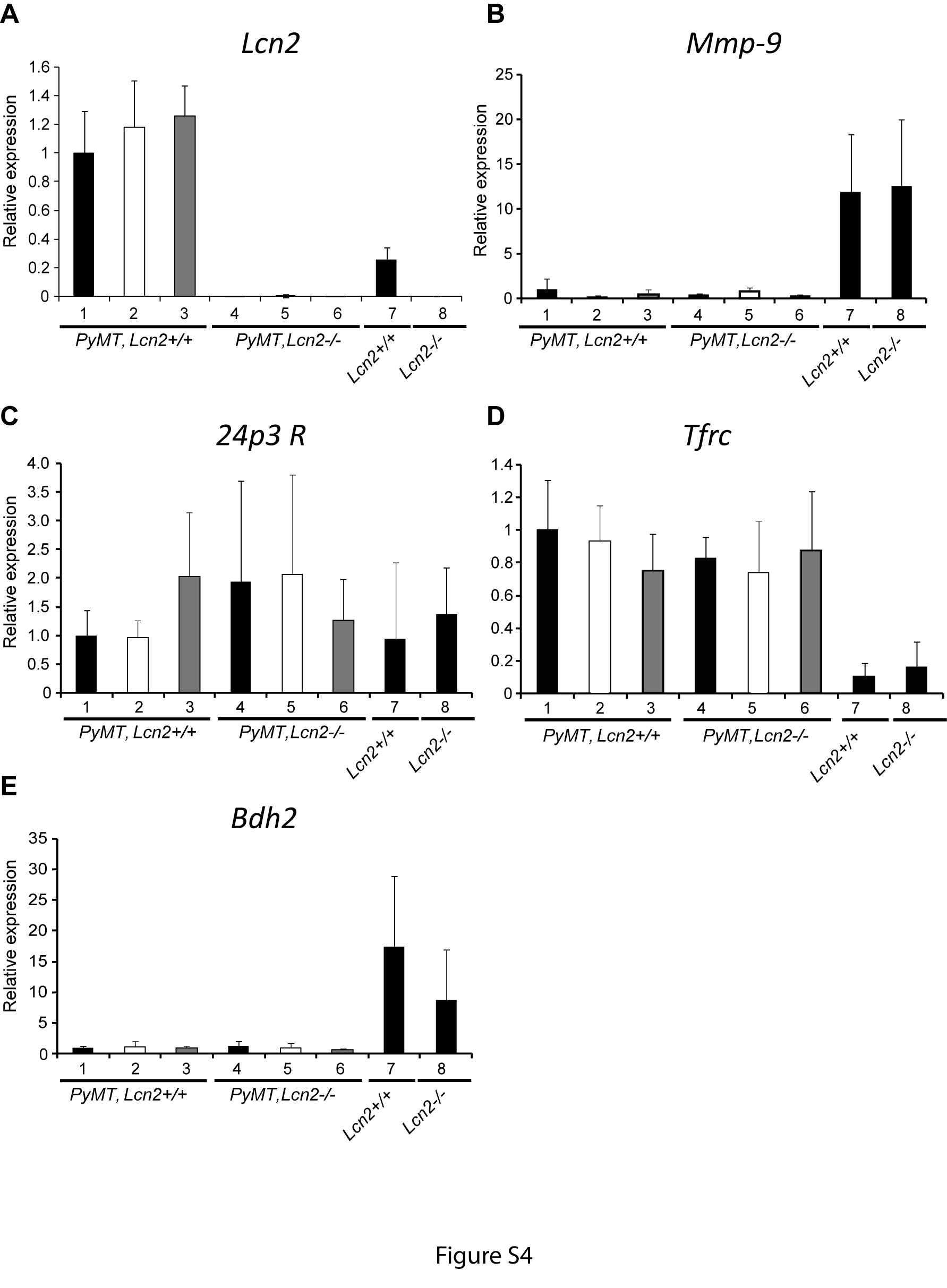

Supplement: Figure S4 — Quantitative real-time PCR analysis of mRNA levels in mammary tumors/glands. A, Lcn2. B, Mmp9. C, 24p3R. D, Tfrc. E, Bdh2. The vertical error bars represent standard deviations. The mean expressions are shown in three PyMT, Lcn2+/+ mice with small tumors (1), three with intermediate size tumors (2) and three with large tumors (3), and the mean expressions are shown in three PyMT, Lcn2-/- mice with small tumors (4), three with intermediate size tumors (5) and three with large tumors (6) located to the fourth mammary gland on the left hand side. The PyMT negative mice were selected randomly and number (7) and (8) represent two mice each. Expression levels of each marker are shown relative to the value found in small tumors of PyMT, Lcn2+/+ mice, which is given the value 1. Tumor volumes: 1) 0.07 cm3, 0.15 cm3, 0.17 cm3 2) 0.47 cm3, 0.55 cm3, 0.68 cm3 3) 0.82 cm3, 0.98 cm3, 1.50 cm3, 4) 0.02 cm3, 0.08 cm3, 0.09 cm3 5) 0.50 cm3, 0.54 cm3, 0.63 cm3 6) 0.73 cm3, 0.98 cm3, 1.33 cm3 Abbreviations: PyMT: MMTV-PyMT. (TIF) [file pone.0039646.s004.tif]
